# ‘That's what makes me better’: Investigating children and adolescents' experiences of pain communication with healthcare professionals in paediatric rheumatology

**DOI:** 10.1002/ejp.2043

**Published:** 2022-10-21

**Authors:** Rebecca Rachael Lee, Danielle Mountain, Mark Connelly, Tonya M. Palermo, Sarah Peters, Lis Cordingley, Janet E. McDonagh

**Affiliations:** ^1^ Centre for Epidemiology Versus Arthritis Centre for Musculoskeletal Research, Division of Musculoskeletal and Dermatological Sciences, Faculty of Biology, Medicine and Health, University of Manchester, Manchester Academic Health Science Centre Manchester UK; ^2^ National Institute for Health Research Biomedical Research Centre Manchester University Hospital NHS Trust Manchester UK; ^3^ Division of Developmental and Behavioral Health Children's Mercy Kansas City Kansas City Missouri USA; ^4^ Center for Child Health, Behavior and Development Seattle Children's Research Institute Seattle Washington USA; ^5^ Manchester Centre for Health Psychology Division of Psychology and Mental Health, University of Manchester Manchester UK; ^6^ Royal Manchester Children's Hospital Manchester University Hospitals Trust Manchester UK

## Abstract

**Background:**

Pain communication should be an integral part of clinical consultations, particularly in paediatric rheumatology where children and adolescents frequently present with chronic musculoskeletal pain. To date, literature exploring the nature of and extent to which pain communication occurs has focused on healthcare professionals as respondents, yielding inconsistent and incomplete findings. The aim of this study was to explore children and adolescents' experiences of pain communication in the context of paediatric rheumatology consultations.

**Methods:**

Data were collected using semi‐structured telephone interviews with children and adolescents recruited from three tertiary paediatric rheumatology centres in the United Kingdom. A framework analysis approach was used to explore the similarities and divergences in participant accounts.

**Results:**

Twenty‐six children and adolescents (aged 6–18 years, median = 14, 58% female) participated. Diagnoses included: juvenile idiopathic arthritis, Chronic Idiopathic Pain Syndromes, Ehlers Danlos Syndrome/Hypermobility. Four themes were identified: (1) Co‐ordination of pain communication; (2) Barriers to pain communication; (3) Facilitators of pain communication; (4) Dissatisfaction with pain communication. These themes particularly encompassed the process of communication, disclosure of effective and ineffective approaches and the impact of communication. Participants expected questions about pain, felt cared about and found talking about pain natural. Challenges included augmenting the feeling of being different to peers and concerns about management plans changing as a result of pain conversations.

**Conclusions:**

Children and adolescents recalled a range of effective and ineffective pain communication approaches. Our study informs recommendations which highlight how healthcare professionals can improve their communication about pain with children and adolescents in the future.

**Significance:**

Our findings demonstrate that children and adolescents attending paediatric rheumatology expect to be and value being asked about their pain during consultations with healthcare professionals. Children and adolescents remember many of the processes involved, experiences of and the outcomes of pain communication. The current study reveals insights which can improve healthcare professional pain communication with children and adolescents. Our study introduces key recommendations for healthcare professionals to have more effective pain conversations in future.

## 1 INTRODUCTION

Paediatric rheumatology receives a wide range of referrals for which chronic musculoskeletal pain is the main presenting concern (Clinch & Eccleston, 2009; Davies & Copeman, 2006; Kimura & Walco, 2007; McGhee et al., 2002). Children and adolescents' experiences of chronic musculoskeletal pain can vary widely along multiple dimensions such as the location, intensity and quality of pain; moreover, there may be associated impact of pain on both physical function and emotional well‐being (Edmond & Keefe, 2015; Huguet et al., 2010; Khanom et al., 2020; Schanberg et al., 2003). Comprehensive and developmentally appropriate assessment and communication about pain features are essential for validating an individual's report and experiences of pain (Defenderfer et al., 2018; Lang et al., 2018), as well as for informing treatment approaches and achieving optimal outcomes (Hadjistavropoulos et al., 2011; Hirschfeld, 2014).

In UK paediatric rheumatology settings, some healthcare professionals perceive the assessment and communication of chronic musculoskeletal pain in children and adolescents to be hindered by time, resources and constraints in healthcare professionals' training in how to ask questions about and address paediatric pain (Lee et al., 2020; Lee et al., 2021). However, other research investigating healthcare professionals' perspectives (particularly nurses) has found that when pain communication does occur in clinical practice, healthcare professionals believe these conversations help to contextualize pain, educate and empower patients and support patient and family coping with pain (Jordan et al., 2021). Pain education in paediatrics (communicating about and teaching a child/adolescent about the underlying biopsychosocial mechanisms of pain, ultimately leading to modifications in their concept of pain) has been found to be a key component of effective multi‐disciplinary pain management (Harrison et al., 2019). Validating children's and adolescents' pain experiences in this way has been specifically associated with improved pain outcomes.

Research exploring the perspective of children and adolescents about healthcare communication, particularly about chronic pain, is limited (Beresford & Sloper, 2003). Some literature has highlighted that children and adolescents believe that healthcare professionals do not understand their pain and healthcare professionals rarely provide them with strategies on how to manage pain in specialized chronic pain programs (Dell'Api et al., 2007). Children and adolescents with juvenile idiopathic arthritis report that it can be challenging to engage directly in conversation with healthcare professionals, often relying on parents to relay important information in general to healthcare professionals (Lundberg et al., 2021).

Further research exploring pain communication in paediatric rheumatology practice is needed, given the inconsistent and incomplete perspectives in extant literature and the prominent role that pain has for children and adolescents being managed in this setting. Therefore, the aim of this study was to investigate children and adolescents' experiences of pain communication from their history of interactions with healthcare professionals in paediatric rheumatology in the United Kingdom. In particular, we were interested in children's and adolescents' experiences of key stakeholders in pain communication (e.g., which healthcare professionals communicated about pain and the role of parents in these conversations), children's and adolescents' insights about the structure and content of these conversations as well as the appropriateness of pain communication styles from children and adolescents' perspectives.

## 2 METHODS

### 2.1 Design

This study was a qualitative semi‐structured telephone interview study with children and adolescents. The study has been structured in accordance with the consolidated criteria for reporting qualitative research (COREQ) (Tong et al., 2007) (Please see Appendix S1 for a completed COREQ checklist).

### 2.2 Participants

Participants were recruited from specialist paediatric rheumatology centres at three tertiary paediatric hospitals across the United Kingdom. Children and adolescents were considered for inclusion if they were aged between 5 and 19 years of age and were under the care of the paediatric rheumatology team and able to communicate in English. Exclusion criteria included children and adolescents who were within 3 months of discharge from the paediatric rheumatology service, being transferred to adult services and not being able to communicate in English. There were no exclusion criteria based on cognitive impairment. A purposive sample (*n* = 118) of children and adolescents of different ages, different conditions and different durations of illness were approached for participation in the study to ensure a diverse sample of experiences and perspectives were captured.

### 2.3 Procedure

Ethical approval to conduct this study was provided by the East Midlands Nottingham Research Ethics Committee (20/EM/0195). Eligible participants were identified from clinical databases by healthcare professionals within the paediatric rheumatology team and clinical research nurses. Written information about the purpose and procedures of the study was sent directly to potentially eligible children and adolescents (and their parents). Alternatively, healthcare professionals and clinical research nurses discussed the study with eligible children and adolescents during/immediately after their clinical consultations. These discussions were structured with standardized information which had been provided to healthcare professionals by the research team. Interested participants were asked to return a reply slip in a pre‐paid envelope to the research team.

The lead researcher (RRL), a female postdoctoral researcher with experience in conducting qualitative research with families and healthcare professionals, contacted interested children, adolescents and parents to discuss the study further. The researcher had no contact with children and adolescents prior to this study. For children and adolescents who wished to participate in the study, a convenient date and time for a telephone interview were arranged. Informed consent from parents and assent from children and adolescents under 16 years of age was audio‐recorded before the interview took place. For participants aged 16 years or older, informed consent was provided by the adolescent themselves and audio‐recorded in the same way before the interview began.

All interviews with participants were conducted by the lead researcher (RRL) between April and October 2021 (during the COVID‐19 pandemic). Social distancing guidelines were in place during the data collection period for this study, therefore telephone interviews were conducted. Telephone interviews are considered to be an acceptable and valuable mode of interviewing, arguably yielding as rich and reliable data as interviews conducted face‐to‐face (Sturges & Hanrahan, 2004). Telephone interviews have several advantages which were particularly useful in the context of interviewing children and adolescents. For example, telephone interviews are useful for engaging with hard‐to‐reach groups which were particularly pertinent to the current study in which the researcher required access to children/adolescents via their parents or guardians. Furthermore, telephone interviews were viewed as more feasible and time‐efficient by participants. For the current study, participants were not required to travel to interview locations and interviews could be more easily co‐ordinated around school and other commitments. It has been found that telephone interviews can be viewed as contributing to a stronger sense of anonymity by participants. This was clearly apparent in the current study where we found that children and adolescents talked freely and openly with the researcher during the interviews.

At the beginning of the interviews, children and adolescents were asked to provide information about their date of birth, gender, diagnoses, age at diagnosis (of the condition for which they were referred to paediatric rheumatology for) and medications. Where children and adolescents were unable to provide this information, the interviewer liaised with parents to capture these details. Following this, children and adolescents were asked to provide information about their interactions with healthcare professionals within the paediatric rheumatology team, including how many healthcare professionals in total they saw from the team, which healthcare professional they saw the most frequently, and which healthcare professional they believed talked to them the most about pain during their consultations. The interview topic guide was based upon an earlier study by the research team which explored healthcare professional perspectives on pain assessment and communication in paediatric rheumatology (Lee et al., 2019). The interview schedule was initially drafted by the lead researcher (RRL) and refined through meetings with the study team. There was additional direct input from children and adolescents from patient advisory groups (specifically YOURRHEUM which is a young person's advisory group for those with rheumatic conditions, https://yourrheum.org/), charities and individual patient collaborators on the project (see Table 1 for final interview schedule). This involvement led to changes in the specific wording and order of the interview questions asked but not in the main topic areas covered by the interview topic guide.

**TABLE 1:** Final interview schedule

Questions for children and adolescents
Could you tell me what it is like coming to hospital in the paediatric rheumatology department and how long you have been coming here for?
2 Could you tell me how pain affects you with the diagnosis that you have?
3 What makes your pain better and/or worse?
4 What type of things do the professionals talk to you about when you come to paediatric rheumatology and how much of your appointment is spent talking about pain?
5 How do professionals start conversations about pain with you and how do those conversations go?
6 What type of things do they ask you about pain and do they ask you every time you see them?
7 What would you like professionals to ask you about your pain and how could they best do this in the future?
8 Do you think the conversations you have with all of the professionals that you see are the same/different and how?
9 What do professionals at the hospital say to do when you tell them you have pain?

At the end of the study, participants received a study debrief sheet. A letter of participation for participants was sent to hospital sites for storage within their clinical notes. All children and adolescents who took part in the study were provided with a £20 shopping voucher and a certificate of participation in the study. All semi‐structured interviews were audio‐recorded using an encrypted audio‐recorder and transcribed verbatim for analysis.

### 2.4 Data analysis

A framework analysis approach was used to understand the similarities and divergences in experiences of participants (Ritchie & Lewis, 2003; Ritchie & Spencer, 1994). This analytical approach to data was selected after consideration to other approaches as framework analysis allows multi‐disciplinary teams of researchers to manage, interpret and reduce large data sets, whilst still retaining a holistic and comparable overview of themes across and within the entire data set (Gale, 2013). The theoretical underpinnings of this approach originated from social policy research but it has become increasingly advocated for use in medical and health research because of these advantages. Two authors (RRL and DM) were the main data analysts. DM was a PhD student at the time of the study being conducted (MSc, female) and had experience of conducting qualitative research. NVivo version 12 (QSR International, Warrington, UK) was used to facilitate qualitative data analyses.

Consistent with recommended procedures for framework analysis, our analytical approach to the data involved: (1) familiarization, (2) coding, (3) identification of a thematic framework, (4) indexing, (5) charting and (6) interpretation (Ritchie & Lewis, 2003; Ritchie & Spencer, 1994). These procedures were performed in a non‐sequential order, with the researchers going back and forth between the steps throughout the analysis of data.

In the familiarization stage (step 1), the two main data analysts read/re‐read interview transcripts and listened/re‐listened to audio‐recorded interviews. After familiarization, both analysts began coding the transcripts and created reflective notes about data and codes independently (step 2). In step 3, these codes were used to inform and build a written ‘working’ thematic framework which was developed based upon a priori aims/questions (deductively) and emerging patterns of experiences and perspectives (inductively) from the participant accounts which were being coded. At this point, the framework was described as a ‘working’ framework as further iterative coding, interpretation and re‐coding of data where appropriate was fed back into the framework so it became more exhaustive and robust over time. The ‘working’ framework was discussed amongst the research team until clarity and consensus were gained about the initial themes and codes identified. In step 4 of the analysis, the framework was then applied to the sorting of data, with relevant fragments of the data indexed in NVivo according to the themes outlined in the framework. During this indexing phase, there was even further refinement of the initial framework during which new themes and sub‐themes were identified by the two main data analysts and fed back in to the framework again. In step 5 of the analysis, indexed data were transformed into a chart, which consisted of columns (themes as identified in the framework) and rows (participant interviews) complete with analytical summaries added by the two analysts throughout columns and rows. Please see Appendix S2 (Framework analysis charting process of participant quotes within and across themes) for the full indexing and charting process. Once the chart was complete across all themes and interviews, the two analysts looked across all summaries to interpret the data (step 6 of the framework analysis). Connections between themes and participant accounts were summarized into narratives.

Throughout all of the steps involved in the framework analysis, the analysts (RRL and DM) each kept a reflexive journal independently to keep account of their philosophical standpoints, their ongoing thought processes about the data and any of their potential biases which they felt were influencing the interpretation of data. This is in line with recommended techniques to establish transparency and enhance the trustworthiness in the identification of qualitative themes (Lincoln & Guba, 1985). Broader analytical discussions about the interpretations of data included consideration to these reflexive accounts amongst all members of the research team. The themes retained and presented were decided upon by consensus reached across all members of the research team who were in agreement about the relevance and importance of the themes in light of the research question posed in the study. There were no retention criteria with regard to the endorsement of themes or sub‐themes by a specified number of participants.

The research team decided a priori to the interviews and analysis being conducted that data collection would stop when the data that were being collected were repeating what had been expressed in participant accounts already captured in the study. This is a concept called ‘data saturation’. Data saturation occurs when any further data collection is considered to be unnecessary as no new additional data are being identified which could further inform the themes and sub‐themes (Saunders et al., 2018). In the current study, the point of data saturation occurring was initially determined by the two researchers who were analysing the data (RRL and DM). The analysts' perceptions about data saturation being reached were discussed amongst all members of the research team, to ensure that all research team members were in agreement with this before data collection ended. Data collection ended after 26 interviews when the whole research team was in consensus that data saturation had occurred.

## 3 RESULTS

### 3.1 Participant recruitment and sample description

The final study sample of 26 study participants had a median age of 14 years (Range = 6–18 years) and a median duration of illness of 3 years (Range = 1–11 years) (See Table 2). Twelve children/adolescents were recruited from one hospital, and seven were recruited from each of the other two hospitals involved in the study (*n* = 14 combined). The hospitals have not been specifically named to protect the anonymity of the children/adolescents and healthcare professionals involved in the study. Diagnoses included juvenile idiopathic arthritis (JIA, *n* = 16), Chronic Idiopathic Pain Syndromes (CIPS) (including Chronic Regional Pain Syndrome [CRPS], *n* = 5) and Ehlers Danlos Syndrome (EDS)/hypermobility (*n* = 5). Interview times ranged from 14 to 51 min (Mean = 18.73 min). Participants reported having consultations with between one and six different healthcare professionals in the paediatric rheumatology team. Participants reported the most frequent contact with rheumatologists (69.23%). Rheumatologists were the healthcare professionals with whom children and adolsecent's were most likely to discuss pain with (53.85%) (See Table 3).

**TABLE 2:** Participant demographics

Characteristics			Values
Age at data collection (years), median (IQR)			14.93 years (IQR 11–16)
Female, *n* (%)			15 (57.69%)
Diagnosis, *n* (%) *some patients had multiple musculoskeletal diagnoses	Juvenile Idiopathic Arthritis (JIA)	Oligo	4 (15.38%)
		Poly	9 (34.62%)
		Enthesitis‐related	3 (11.54%)
	Chronic Idiopathic Pain Syndromes (CIPS) (including Complex Regional Pain Syndrome)		5 (19.23%)
	Ehlers–Danlos Syndrome (EDS) and/or hypermobility		5 (19.23%)
Duration of condition (years), median (IQR)			3.83 years (IQR 2–7)
Medications for musculoskeletal condition, *n* (%)	No medications		3 (11.54%)
	Non‐steroidal anti‐inflammatory drugs (NSAIDS)		10 (38.46%)
	Corticosteroids		1 (3.85%)
	Disease‐modifying antirheumatic drugs (DMARDS)		13 (50%)
	Biologics/biosimilars		12 (46.15%)

**TABLE 3:** Participant descriptions of interactions with the paediatric rheumatology team

Number of Healthcare professionals seen in the paediatric rheumatology team by individual child/young person, mean (Range)	3.04 (Range 1–6)
Healthcare professional seen the most, *n* (%)	
Rheumatologist only	18 (69.23%)
Physiotherapist only	4 (15.38%)
Occupational therapist only	1 (3.85%)
Combined appointments with physiotherapists/occupational therapists	1 (3.85%)
Combined appointments with physiotherapists/occupational therapists and psychologists	1 (3.85%)
Child/adolescent could not remember	1 (3.85%)
Nurse only	0 (0%)
Psychologist only	0 (0%)
Healthcare professional that children/young people perceive to discuss pain the most, *n* (%)	
Rheumatologist	14 (53.85%)
Rheumatologist /physiotherapist equal amounts	4 (15.38%)
Physiotherapist	3 (11.54%)
Child/adolescent could not remember	2 (7.69%)
Nurse	1 (3.85%)
Rheumatologist/physiotherapist and occupational therapist all equal	1 (3.85%)
Physiotherapist/occupational therapist equal amounts	1 (3.85%)
Occupational therapist	0 (0%)
Psychologist	0 (0%)

### 3.2 Themes, subthemes and interpretation

Four overarching themes were identified: (1) Co‐ordination of pain communication, (2) Barriers to pain communication, (3) Facilitators of pain communication, (4) Dissatisfaction with pain communication.

## THEME 1: CO‐ORDINATION OF PAIN COMMUNICATION

### 1.1 Expectation of pain communication

Children and adolescents unanimously agreed that it was important for paediatric rheumatology healthcare professionals to ask them about their pain at some point during their consultations, as is evident in the quotes below. There was an expectation that pain should be addressed in consultations because this was part of the professional's job and how children and adolescents with pain ‘got better’;

“It's important because that's their job, isn't it? Like, I think that's important, so you know what's happening”, Participant 2, 15 year old male, JIA.

“Because that's the thing about pain, it's a question I'm obviously expecting. The consultant has been very, very helpful to me and I understand that speaking about my pain is the best way to get help. So, I am as comfortable as I can be speaking about it because I understand it is really important to my treatment”, Participant 18, 16 year old female, JIA.

“Because that's what makes me better”, Participant 19, 6 year old female, JIA.

### 1.2 Purpose of pain communication

As described in the following quotes from the perspective of children and adolescents, the purpose of pain conversations was so that the healthcare professional was able to see how pain had changed, decide what the best treatment was and to investigate any progress with treatments since their prior consultation;

“Because if they ask you how the medication's affecting you and how you are feeling, then they'll be able to work out whether it's a good thing that you're on the medication or a bad thing, they'll know like what other things you need”, Participant 25, 15 year old male, JIA.

Participants also talked about how their pain reports were important for informing healthcare professionals on how to help other children and adolescents with similar pain presentations in the future;

“So they can help me and so they can help other people, because if they get symptoms that I have for something they might think one day when someone else comes in, like, oh, she used to have this, could follow up on that.”, Participant 24, 13 year old female, EDS/Hypermobility.

It was important to children and adolescents that they could tell healthcare professionals everything about their pain, as all pain information had an important purpose to communicate with them;

“I tell them how bad my pain is and I try to tell them in‐depth what it's like and stuff. I'd say that all pain is important. I think it's very important because if they don't know what sort of pain I'm in they might suggest the wrong thing and the wrong treatment and that could make things worse”, Participant 13, 14 year old female, JIA.

### 1.3 Mixed roles and values in parents' pain reporting

Children and adolescents had mixed perspectives on the role and value of their parent's involvement in pain communication with healthcare professionals, as demonstrated in the range of quotes provided below. Some children and adolescents reported that healthcare professionals directed questions about pain to their parents. Some children and adolescents viewed this as problematic, creating particular difficulties for those who did not want to reveal the full extent of their pain to their parents;

“But they were asking all the questions to my mum and I was sat there thinking this is my appointment, it should be me speaking. My mum doesn't know the pain that I'm in. She can see the pain that I'm in but she can't feel the pain I'm in. And then I stopped letting my mum come to appointments with me because it started to turn out like I'd be sat there in the corner whilst my mum was having a consultation with the doctors. I show my mum what I want…I hide my pain very well. So the doctor could be sat there asking my mum how I've been and mum's been like, yeah, yeah, she's fine, she's fine, she's not complained much about it this week, but realistically I could have”, Participant 3, 18 year old female, EDS/Hypermobility.

For other children and adolescents, parents were the main person they told about their pain, more so than healthcare professionals, which made parents valuable advocates in pain reporting, as described below;

“Yeah, my mum always comes in….I think my mum talks about it more than I do because you'll get more out of my mum than you do out of me… sometimes they'll ask me, but if they want to get anything out properly, they'll ask my mum…I'll be like, I'm fine, or something like that, and then my mum will be like, you're not fine…If they want to know about how bad the pain has really been, they know to ask my mum and not me. Because I'll tell my mum but I won't tell them”, Participant 15, 16 year old female, CIPS.

As the below quote suggests, children and adolescents highlighted how parents could describe an outside perspective about pain, which was useful for identifying where the child/adolescent had been struggling but not noticed these difficulties themselves;

“Sometimes I don't notice like my walking and stuff, so she'll describe like if my walking's been bad or something like that…I think my mum talks more than me…my mum gets more worried about like if she notices something that I don't notice. So it's more like smaller things that she's picked up on that's starting to occur”, Participant 22, 16 year old male, CIPS.

Parents were also key for reminding children and adolescents to report pain episodes they had forgotten about;

“Yeah, if I've forgotten something like, cause I'm quite forgetful sometimes. But that…other than that she just leaves me to talk to them.”, Participant 10, 13 year old female, JIA.

### 1.4 Methods in pain communication

Children and adolescents referred to being verbally asked to rate their pain from 1 to 10 or to rate their pain upon a body manikin tool which was used by a range of healthcare professionals. Participants talked about the difficulties of using these tools, as the pain they were asked to reflect upon may have changed in the past week, as evident in the following quotes;

“When you're kind of in the waiting area to go for your appointment, they give you the sheets of paper to answer how you've been doing. They kind of give you like a scale to answer how much pain you've been in. But the only thing with those is it's how you've been feeling for the past month or past week, which I find quite hard because sometimes I could be feeling quite bad one day but then good the next”, Participant 11, 12 year old female, JIA.

“Then she'll bring out this piece of paper with like a body on it, and then I'll tell her where my joints have been hurting and everything”, Participant 25, 15 year old male, JIA.

Other participants mentioned ‘surveys’ or ‘quizzes’ such as the ‘smiley faces’. Participants talked about how pain ratings using these tools would be returned without being asked for further elaboration from the healthcare professional;

“Like one to ten, things like that… I think they write it down and put it in (the clinical notes)  and that's it. I know the consultant used to have a little stick man and label where the pain was…They'd go into more depth, of how you're feeling and how it's affecting you and things like that or if it's affecting anything else in your life”, Participant 16, 16 year old female, JIA.

### 1.5 Specific questions asked about pain

Remembering how pain had been in the past and breaking pain down into its components to tell healthcare professionals about was seen as difficult when broad questions about pain were asked, as demonstrated in the following reflection;

“What type of pain are you feeling, is it niggly pains, is it dull, where have you been feeling those pains. So, it's always how have you been coping in college with your pain. I get a lot of stuff like that. Or how have you been, how has it affected your schoolwork. I get a lot of that. Sometimes I wish there was kind of a way of being able to break it down a bit easier to explain it…I know it has to be asked, how has your pain been, but sometimes that's such a broad question, my brain starts thinking of everything”, Participant 7, 18 year old female, CIPS.

Participants described a range of specific questions they were asked by healthcare professionals about pain, including questions about potential pain causes or pain triggers, pain location, pain qualities, pain frequency, pain timing, pain interference with activities (particularly schoolwork), pain coping, pain changes and pain management strategies tried;

“He'll ask what type of pain it is, and how often, and what time it comes and if there's a point where it gets worse during the day, and how I'm getting on with the medication that I'm currently on. Yeah…It'll just be like, oh, so like, it's just the same questions”, Participant 12, 15 year old female, CIPS.

“She just asks like what kicks it off, how do I solve it, stuff like that…She asks like is there any certain things that set it off or is there like any movements that you find difficult when it sets of”, Participant 22, 16 year old male, CIPS.

## THEME 2: BARRIERS TO PAIN COMMUNICATION

### 2.1 Appropriate timing of pain communication

Children and adolescents found that they were asked about pain straight away during consultations, when they would have preferred to take their time building up to questions about pain, as described in the below participant accounts;

“So, sometimes it is just me dragging myself there and it's kind of I know I just want to be in bed right now. So, bringing up the things that's making me in pain, I don't want to sort of be hit with it straightaway. I kind of want to take my time with it”, Participant 7, 18 year old female, CIPS.

“It's definitely the professional. It's like the first thing that's asked when I come and sit down”, Participant 18, 16 year old female, JIA.

Participants explained that questions about pain were predominantly asked about during physical examinations conducted by healthcare professionals. A repercussion of being asked about pain during physical examinations involving manipulation of joints meant that children and adolescents could be in pain as a consequence of the examination;

“It always starts with the professional. I was doing my exercises and she could see they were starting to be really hurting me, even though I have to do a certain amount so she could analyse me”, Participant 9, 18 year old female, EDS/Hypermobility.

“I think, sometimes, they ask me to do stuff like move my foot around and does that hurt?”, Participant 14, 8 year old male, JIA.

For children and adolescents under the care of several specialities, it could become tedious repeating the same information about pain between different specialities seen at similar time intervals, as highlighted below;

“Sometimes it can be a bit tedious like if I'm seeing orthopaedics and then I'm going to rheumatology and I'm kind of having to repeat myself, but I know it's got to be done”, Participant 7, 18 year old female, CIPS.

Participants were frustrated when they felt that healthcare professionals had not given them the time to say what they wanted to say about pain before leaving their consultation;

“I do occasionally (write a list), but then sometimes I think it's like, not worth it anyway…sometimes if you do it, and they don't as, then it just feels even worse, you know what I mean? Because you had all this you wanted to say, and you never got to say it”, Participant 12, 15 year old female, CIPS.

It was important that children and adolescents did not feel like the healthcare professional was not listening, as this could be perceived as though they were uninterested;

“They ask me first…It feels like they are trying to talk about something different and they are not like that interested”, Participant 17, 10 year old male, EDS/Hypermobility.

It was also important that children and adolescents did not feel forced to provide information about pain if it was something they did not want to talk about that day;

“I don't want to talk about this because when you talk about the pain, it's in the front of your mind. You have to think about it”, Participant 7, 18 year old female, CIPS.

### 2.2 Difficulties finding the terminology to express pain

Children and adolescents sometimes did not know how to describe their pain and they were unsure about what terminology to use in asking or answering healthcare professionals' questions about pain, as discussed in the participant reflections below;

“But then she just starts talking to my mum…about stuff I don't know and words I kind of know, but not much”, Participant 14, 8 year old male, JIA.

“I didn't really know how to answer them because I didn't really know how to describe it, if you know what I mean… I don't really know how I felt…I mean, sort of, like, what sort of pain is it, I'm like, I don't know, it just hurts”, Participant 16, 16 year old female, JIA.

For other children and adolescents, talking about pain had become normal to them and in these instances, they found it easier to have conversations about pain with healthcare professionals. As can be seen in the quotes below, participants explained that with age, they were able to understand how to talk about their pain better and developed more confidence with pain discussions;

“I mean obviously because I was a child it was a lot harder to explain the pain, because I didn't really understand it… But those questionnaires they were really helpful…I mean obviously I've been doing it for quite a long time, so I'm used to it… I think it's because I used to see her a lot more and she seemed really, really supportive”, Participant 20, 17 year old female, JIA.

“To me it's a normal everyday thing… if I had to mention it to people that weren't doctors or people that weren't my mates I'd be okay…it's easy to talk about”, Participant 24, 13 year old female, EDS/Hypermobility.

### 2.3 Feeling nervous, scared and/or overwhelmed

Participants talked about how they sometimes felt ‘nervous’ and ‘scared’ to report pain to healthcare professionals;

“Sometimes I do get a tiny bit nervous… but never full on I don't want to tell you, but sometimes…I've never been like that…I think it's more I'm just a bit scared or something like that, I think… Like, I know I'm not scared, but I just feel weird inside…Like butterflies in your tummy”, Participant 14, 8 year old male, JIA.

These feelings appeared to arise from concerns about possible additional or new treatments including new medications, additional exercises to do, or additional referrals and investigations that might find ‘something else’ wrong with them, as demonstrated in the following quotations;

“Because if I tell the physiotherapist, then she'll just make me do loads of exercises. I think just sometimes if I'm just not in the mood, I will just not be in the mood and I won't mention it, I'll just keep it to myself”, Participant 1, 17 year old male, JIA.

“There's times when I've not wanted to bring up my pain…because I didn't want the stress of knowing that possibly I could have something else wrong with me. Do you get what I mean? The ways they ask me is obviously good, because they don't force anything out of me like, they don't get frustrated or thingy if I have a little emotional tic…Because when I first started to go I didn't want to accept that I had anything wrong with me. I didn't want to accept that I was poorly or that was me”, Participant 3, 18 year old female, Hypermobility.

Talking about pain confirmed feelings of difference in relation to peers, as described in the quotes below;

“Because it's not very nice, it's…it's not very nice. Sometimes because you don't want to talk about it because it makes you feel different. Because normally, people of my age, you don't really have to say if you've been feeling well, if you've been hurting or not. So it kind of just makes you feel quite different from everyone else”, Participant 11, 12 year old female, JIA.

“I don't like speaking about the pain…I think it's just because I know I'll start tearing up because then it just makes me feel like I can't do what other people can do”, Participant 15, 16 year old female, CIPS.

Pain conversations were also viewed as worrisome to some participants as it reminded them that their pain was going to affect them for the rest of their lives;

“I was quite scared to be honest. It was like thrown in the deep end and I didn't really understand what it was…I didn't even realise children could get arthritis, so when they were asking me all these questions it was like oh it's quite scary because it's going to affect my life…I mean that was the one thing I was really worried about because I feel…I know when I'm older I'm probably going to have to have a joint replacement. I can already feel my joints grinding. So that always was on my mind”, Participant 20, 17 year old female, JIA.

Children and adolescents reported sometimes generally feeling overwhelmed with talking about pain because they were tired of feeling and thinking about pain, as demonstrated in the below quote;

“They are fine questions. They've changed depending on how better or worse the pain has got…Because I'm fed up of my pain and I just don't really want to talk about it”, Participant 17, 10 year old male, EDS/Hypermobility.

### 2.4 Pain uncertainty

Participants talked about the difficulties of managing pain uncertainty. These difficulties could be exacerbated through communication about pain with healthcare professionals, particularly when negative test results were provided;

“Well, I'd wish that I could tell them the pain, where it is, and they could snap their fingers and give me a diagnosis…Because with me it's all the uncertainty that gets me wound more than anything. I wish that I knew what was going on with my body, but unfortunately I don't”, Participant 3, 18 year old female, EDS/Hypermobility.

“Not with sort of the departments I've been seeing recently. I felt like sometimes when I started going and I was with orthopaedics, it was sort of that…It was sort of that, well, your x‐rays are not really showing anything, your MRI is showing a bit. And then you say, but I'm in pain”, Participant 7, 18 year old female, CIPS.

Participants were cognisant about the impact that this uncertainty may have had on the healthcare professionals who were trying to talk to them about pain, suggesting that healthcare professionals may have felt ‘helpless’ at times;

“That's really all they could do to control the pain…Saying that you feel pain and they don't know what to do, it might make them feel a bit helpless”, Participant 20, 17 year old female, JIA.

Participants tried to manage their own uncertainties by not thinking too much about what ‘might’ happen, as demonstrated in the following quote;

“Well, really, when I was younger, I was quite confused, but now I'm just, like, that's my arthritis, I won't worry about it because I mean, I know it's not the end of the world now that I've got it…But I used to think bad stuff about it, but now I'm…just wait and see if this will happen because there's no point thinking in my mind, oh, this is going to happen when you don't know that it's going to happen”, Participant 14, 8 year old male, JIA.

### 2.5 Pain dismissal

Participants felt as though pain was less of a focus in consultations when pain was low or was not believed to be related to their arthritis. They talked about feeling like pain was ‘brushed off’ when it was referred to by the healthcare professional as mechanical pain, even though to children and adolescents these pains felt the same and had the same impact, as seen below;

“I feel like they're less focused on it…to me, it's the most important thing…I don't think is it the arthritis pain or is it this pain or is it that pain, I just think my leg's hurting…I think they refer to the other one as a mechanical pain and the arthritis one as arthritis pain. So the word mechanical… Puts metal in my mind… I think sometimes it could have been dismissed even though…I could be feeling a lot of pain and it could be affecting me in my day to day, but if there's no signs of inflammation or there's no swelling that they can see, then it sometimes got brushed off”, Participant 1, 17 year old male, JIA.

Participants talked about pain being dismissed when investigations showed no cause for pain or when pain was occurring when the disease was in remission;

“Maybe if I'm in remission or not…for the last few appointments. I find it unlikely given my condition. It's known for pain, that's what I'm there for really”, Participant 8, 13 year old female, JIA.

Some participants felt that healthcare professionals did not listen to them about their pain and instead, the healthcare professional's response would be to close down conversations about pain in order to talk about something different;

“It feels like they are trying to talk about something different”, Participant 17, 10 year old male, EDS/Hypermobility.

Participants talked about how professionals could sometimes make it feel as though they were exaggerating their pain, even though children and adolescents recognized that healthcare professionals might not have intentionally meant to make them feel this way;

“Because obviously I'm not making it up, but sometimes I feel like, they don't mean to do it, but sometimes the way some of them can talk to you, makes you feel like it's not as bad as you're making it out to be. Yeah…I mean sometimes it's upset me”, Participant 12, 15 year old female, CIPS.

Some children and adolescents had been told to ‘push the pain away’ by not thinking about it, as described below;

“One of the coping mechanisms we've been kind of using is pushing the pain away. Yes. On those bad days you're just like I don't even want to be here”, Participant 7, 18 year old female, CIPS.

## THEME 3: FACILITATORS OF PAIN COMMUNICATION

### 3.1 Informal conversations

It was important that healthcare professionals asked about things other than pain during the course of the consultation, such as hobbies, as this created a ‘friendly environment’;

“We'll have a five or ten minute conversation about like what we've done on the weekend and everything. Feels dead comfortable that. She always asks about my music every time I come in… So it's a really friendly environment… And I know that the rheumatologist is here to help me and I've got a bond with them we've formed a relationship”, Participant 3, 18 year old female, EDS/Hypermobility.

Being asked about other things distracted children and adolescents from questions focused on pain, which they found valuable, as described in the following quote;

“She'd ask me how was the pain, but other than that, it was mostly how I am around the house, or how is…? So it wouldn't be focused on just the pain, she'd try and distract me from other things, from just talking about my hip and the pain…She'd ask me how do I think that the pain is triggered or stuff like that”, Participant 15, 16 year old female, CIPS.

### 3.2 Feeling reassured and cared for

Participants spoke about the importance of feeling reassured when they reported pain and its impact to healthcare professionals, with reassurance particularly being provided that being affected by their pain was acceptable;

“Sometimes I just want to be told it's okay to have a bad day. It's okay to not want to go out with your friends if you're not feeling too great in yourself. Because I know that I'll try and push myself and I'll just make myself feel worse”, Participant 6, 9 year old female, CIPS.

Being asked about pain gave children and adolescents an opportunity to offload about their experiences and made them feel that professionals cared about them which was comforting to them;

“I think it's really important because I think it shows that they care. And it is nice to be asked how I've been coping or how much pain I'm in because I think it just gets it off my chest really. “Cause when I'm in pain I don't really say anything”, Participant 8, 13 year old female, JIA.

For some children and adolescents, not feeling like a burden was important as they did not want to feel as though they were a hindrance to healthcare professionals by reporting pain;

“I just don't like bothering people… I didn't really want to bother anyone with it. Yeah, like I thought it was unneeded because I didn't think I'd have anything wrong with it”, Participant 23, 12 year old male, JIA.

### 3.3 Familiarity

Familiarity with healthcare professionals within the paediatric rheumatology team was important, as children and adolescents felt that this enabled the professionals to understand their condition better and it could help them to tailor their approaches to pain management, as evident in the quotes below;

“I think as well the team's been able to understand more because they've been able to get to know me more…now it's sort of how do we deal with pain if we were in the middle of a drama lesson. Stuff like that. We've been able to tailor it more”, Participant 7, 18 year old female, CIPS.

“I think it's because she knows me…so there's less crying”, Participant 15, 16 year old female, CIPS.

For other children and adolescents, familiarity with healthcare professionals did not necessarily mean that conversations about pain became easier as it still meant that new questions regarding their diagnosis or prognosis could be identified which could be challenging to experience;

“Because it's like every time I go there's a new question that arises. But the rheumatologist has grown up with me in these past four years. And when I see the rheumatologist I don't feel obliged or anything to talk”, Participant 3, 18 year old female, EDS/Hypermobility.

Other participants found the familiar format of the questions asked during consultations to be redundant because being asked the same questions every time meant that nothing changed;

“Yeah, they ask the same like five questions every time I go in. And, by the time I come out, nothing gets changed on what we're doing…it's a bit of a waste of time”, Participant 21, 12 year old male, EDS/Hypermobility.

### 3.4 Communicating and managing the emotional impact of pain

Several participants talked about the importance of healthcare professionals asking not only about what pain physically limits them from being able to do, but also how pain impacts upon their mental health, as demonstrated in the following interview excerpt;

“You hear it a lot with mental health services…Sort of when it comes to pain, I think the mental health side's forgotten a little bit. I just want to sort of reiterate the fact it affects both physical and mental health, and I think they need to be addressed both. Yes, I think that's just sort of been a growing up realisation. And as well in hospital, I think it was the consultant where I said, oh, I've been having a few bad, down days and she kind of just brought up is it pain bad, down days or is it mental bad, down days. And that's when it sort of… It was almost a lightbulb moment where I thought, oh, my gosh, I can have bad mental health days. That is normal”, Participant 6, 9 year old female, CIPS.

Participants described a variety of ways in which they had learned to self‐manage the emotional impact of their own pain where healthcare professionals had been unable to offer them any advice or solutions. For example, children and adolescents explained how they would try ‘masking’ or ‘distracting’ themselves from pain with other types of pain or activities;

“I just mask it with other pain…even though it wouldn't help me in the long run, it would get me out of it for that moment…there's that, so I just have to push myself”, Participant 1, 17 year old male, JIA.

Accepting that something was wrong with them meant that they were able to deal with the emotional side of pain better. However, children and adolescents talked about hiding the true extent of pain and not dwelling on the fact that there was something wrong with them in their conversations with healthcare professionals;

“I didn't want to accept that I was poorly or that was me. But now that I know what's going on a little bit more I can deal with the emotional side of it a lot better than I could…I'm one of them people that I don't want to dwell on the fact that there's things wrong with me”, Participant 3, 18 year old female, EDS/Hypermobility.

## THEME 4: DISSATISFACTION WITH PAIN COMMUNICATION

### 4.1 Challenges interpreting pain advice

Participants explained how they did not always understand healthcare professional's advice on how to manage pain following communication, as evident in the following quotes. Healthcare professionals often gave mixed messages about pain which were difficult to put into practice: for example, if children and adolescents were doing too much they should do less, and if they were not doing enough, they should do more;

“So if I go out too much, then it would be, like, take it easy. If I don't go out enough, then just do some physio or something”, Participant 2, 15 year old male, JIA.

Participants considered there to be a fine line in knowing their triggers between these two extremes, as described in the following quote;

“She says that to me to keep myself busy but not to overdo it. It's finding that fine line of when it's time to stop distracting myself… They tell me to distract myself, like try not to think about it as much, try not to google it, don't google it, whatever you do, don't google your symptoms”, Participant 3, 18 year old female, EDS/Hypermobility.

Some participants talked about how they had found that nothing made their pain better from advice provided to them by healthcare professionals;

“Nothing actually makes it better. It sort of gets worse by each hour, it gets gradually worse pain”, Participant 6, 9 year old female, CIPS.

### 4.2 Anger at healthcare professionals’ pain management explanations

Many participants talked about the ‘boom and bust’ cycle that healthcare professionals had used in their pain management explanations to children and adolescents. Some participants felt angry with this explanation, particularly when they felt like they had not done enough activity to ‘bust’, but h professionals advised them that they had;

“She'll tell me that I'll know when to stop, but I won't stop because I think that I can do it … Like she's got this thing called bust and booms… I've pushed myself, and other times I haven't, but they think I have. So it just makes me feel that… I don't know if it's anger”, Participant 15, 16 year old female, CIPS.

Sometimes healthcare professionals would talk about how other children and adolescents were managing their pain, which participants did not find helpful for explaining their own pain, as highlighted in the following interview excerpt;

“Just the same over and over again. Just get a hot bath, get some water bottles, take some painkiller. Because she always kept on going on about other people. It would always be like other people are struggling as well, and I'm just like yes, I know that…It's just like every session she'd just go on about other people. It would get really annoying”, Participant 24, 13 year old female, EDS/Hypermobility.

Some participants felt like they were provided with no advice on how to manage pain and if they suggested potential solutions themselves, the healthcare professional would disagree which left children and adolescents own perspectives feeling overlooked, as described below;

“Any ideas I suggest is…is a swift no. Or once, they even said, we'll get back to you, and then in like six months' time they came back and said no…Because it's like, you go in, you talk about why it's so bad and then whenever you give any ideas, they just fob you off”, Participant 21, 12 year old male, EDS/Hypermobility.

## 4 DISCUSSION

The current study explored experiences of pain communication in paediatric rheumatology from the perspectives of children and adolescents with a broad range of long‐term musculoskeletal conditions. Participants provided insight into how pain communication was coordinated, barriers and facilitators to conversations about pain and dissatisfaction with elements of pain communication with healthcare professionals. Children and adolescents could remember many of the processes and outcomes involved in pain conversations, and they highly valued conversations about their pain with healthcare professionals. Many were comfortable directly engaging in pain discussions with healthcare professionals because they expected questions about pain to be asked, felt cared about when asked questions about pain and found talking about pain natural as it had become a normal part of everyday life. Challenges in pain communication identified by participants included augmenting the feeling of being different from peers and concerns about management plans changing as a result of conversations with healthcare professionals.

There has been mixed evidence on whether pain communication routinely occurs in clinical paediatric rheumatology settings (Jordan et al., 2021; Lee et al., 2020; Lee et al., 2021). Importantly, the current study found compelling evidence from the perspectives of children and adolescents to suggest that effective pain conversations are featured in paediatric rheumatology settings. For example, multi‐dimensional pain assessment appears to be commonly done at least informally through asking about potential pain causes/triggers, location, qualities, frequency, timing, interference with activities and/or schoolwork, coping, changes and management strategies. There was also evidence in our study of formal pain assessments being used (such as pain rating scales), although children and adolescents emphasized the limits of these tools in describing their pain to others.

This study extends findings established in previous research about the roles and values of parents in relaying information to healthcare professionals (Lundberg et al., 2021). Similar to our study, Lundberg et al. (2021) found mixed perspectives from children and adolescents about the value of parents in pain communication. In this research, some children and adolescents believed parents provided a useful external perspective and they spoke to them frequently about their pain. Other children and adolescents intentionally or unintentionally concealed aspects of their pain experience from parents, such that parents were unaware of the true extent of their child's pain. This presents a challenge for healthcare professionals. Effective conversations about pain with parents present as stakeholders in these conversations may first require healthcare professionals to explore and evaluate the child's preferences for the role of their parent in pain communication. Preferences may vary based on age and developmental level (particularly the cognitive abilities) of the child/adolescent in reporting their own pain (Emerson & Bursch, 2020). For example, younger children may rely on their parents more than older children to report their pain on their behalf, because they may not have developed the understanding and/or vocabulary to be able to describe pain experiences to a healthcare professional (Chan & von Baeyer, 2016). Most theories of cognitive development posit that it takes up to around 11 years of age to develop the intellectual capacity to process complex information to understand and to then describe complex concepts or experiences such as pain (Caplan & Bursch, 2012). Furthermore, the degree to which healthcare interactions are co‐ordinated according to developmentally appropriate principles will further influence the degree of participation by younger patients (Rapley et al., 2019). Nurturing the child/adolescent skills in self‐reporting and self‐managing their own pain is important wherever possible.

In the current study, children and adolescents described how pain dismissal was a barrier to effective pain communication. These findings contribute to the growing evidence base of pain dismissal occurring during childhood and adolescent healthcare consultations (Defenderfer et al., 2018; Edmond & Keefe, 2015; Igler et al., 2017; Lang et al., 2018). In this study, children and adolescents felt that pain was overlooked when low in intensity or when deemed by healthcare professionals to be ‘mechanical’ rather than due to the underlying rheumatological condition. This occurred despite children and adolescents explaining that these pains felt the same and had the same impact. Pain dismissal has implications for future pain communication as literature suggests that children and adolescents feel a sense of hostility towards the individual who dismissed their pain, which ultimately damages the relationship as they become disengaged and less likely to adhere to recommended management plans (Defenderfer et al., 2018). Dismissal and the subsequent impact on relationships with healthcare professionals infiltrates into and affects other aspects of care. In past literature, adolescents have reported significant negative reactions to pain dismissal and subsequent invalidation of their pain experiences by healthcare professionals, such as depression, anxiety, anger and feeling isolated (Wakefield et al., 2018). However, some children and adolescents in this study appeared to appreciate that healthcare professionals may not have intentionally invalidated their report of pain. Past research from healthcare professionals’ perspectives has found that they can sometimes feel helpless, frustrated and uncomfortable with handling the unexplained nature of pain within consultations (Lefèvre et al., 2019), in line with how children and adolescents interpreted elements of pain dismissal in the present study.

Our findings on children and adolescents' acceptance of healthcare professionals' explanations for pain were similar to themes in Sørensen and Christiansen's (2017) study which highlighted that children and adolescents feel anxious when given conflicting explanations and pain management advice. However, our study found children and adolescents' reactions were more closely related to anger, as opposed to uncertainty. Sørenson and Christiansen's study also found that children and adolescents experienced despair when presented with negative investigation results which highlighted they were different from their peers, which was consistent with children and adolescents' reactions to inconclusive tests in the present study. When presented with the option of receiving psychological support for pain management later on in their care, children and adolescents in both this and Sørenson et al's study felt as though healthcare professionals were reluctant to introduce any further management strategies themselves. An important implication of this finding is that psychological support should be integrated into pain management as early as possible to emphasize the importance of psychological support and reduce later barriers to access.

Findings should be interpreted in light of several study limitations. One potential limitation of the current study was that experiences about pain communication came from children and adolescents who were patients at specialist tertiary paediatric rheumatology centres across the United Kingdom, which had reputations for providing high‐quality healthcare. Thus, findings may not generalize to other settings where children and adolescents with chronic musculoskeletal conditions receive care (e.g., primary or secondary care settings). In addition, a wide age range was included in the sample but there were only a small number of children and adolescents representing each age group, which makes it challenging to compare themes by age or to identify unique developmental preferences in pain communication. Future research should aim to investigate whether there are age and/or developmental specific, or even diagnosis specific, pain communication preferences. Future research should also explore parental experiences and perspectives on pain communication during healthcare consultations, as well as the varying role of parents and the different values placed upon their position (by children/adolescents) within these often triadic healthcare communication encounters. These avenues remain unexplored despite parents being significant key stakeholders in communication during consultations.

### 4.1 Recommendations for the future

The findings of this study highlight a range of effective and ineffective pain communication approaches from the experiences of children and adolescents with chronic musculoskeletal conditions. We propose several recommendations for healthcare professionals communicating about chronic pain specifically (taking into account that there are general communication recommendations elsewhere [Kim & White, 2018]). Recommendations are grouped according to the themes identified;

#### 4.1.1 Co‐ordination of pain communication

- Ask about pain in every consultation, as children and adolescents generally expect this.
- Allow the child/adolescent to settle into the consultation before beginning to ask questions specifically about pain.
- Ask children/adolescents for more than one average pain rating to take into account temporal differences in pain and pain that can be provoked vs unprovoked. For example, healthcare professionals could also ask about pain at different times of the day/week and/or pain before, during and/or after particular activities.
- Break down questions about pain into different components (e.g. ask about location, intensity, qualities and interference individually rather than asking broadly about pain).
- Explore the child/adolescents' preferences for their parents to be included or not included within pain reporting.

#### 4.1.2 Barriers to pain communication

- Avoid forcing information (if appropriate) about pain in circumstances where children and adolescents seem reluctant to discuss pain in detail.
- Avoid verbal or nonverbal cues suggesting frustration or dismissal of pain reports, and instead convey a willingness and commitment of time to discuss pain that the child/adolescent wants to report or expand upon.

#### 4.1.3 Facilitators of pain communication

- Balance discussion about pain with asking about non‐medical topics such as hobbies/interests of the child/adolescent, if time allows.
- Ask children and adolescents about how pain is affecting them emotionally and cognitively, in addition to asking about how it physically limits them.
- Make sure terminology is appropriate to the developmental level of the child/adolescent and make sure that the vocabulary used for pain explanations resonates with their own descriptions and explanations by asking what they understand by particular terms (e.g., the term ‘mechanical pain’ may be problematic).

#### 4.1.4 Dissatisfaction with pain communication

- Provide clear and tailored pain management advice (e.g., what is considered to be too much or too little activity for that child/adolescent).
- Elicit children and adolescents' own perceptions of their limits and their own ideas about how they think they could best manage their pain.

These recommendations will improve pain communication between healthcare professionals, children and adolescents, specifically those with chronic musculoskeletal pain who are managed in paediatric rheumatology settings. However, future research efforts which focus on the translation and mobilization of our findings into real‐world practice are needed. There is a need to address the gap between our improved understanding of paediatric pain derived from research such as this, and current clinical practices (Chambers, 2018). Additional efforts are required to translate research findings and implement evidence‐based recommendations in clinical settings.

## 5 CONCLUSIONS

This study presents a comprehensive overview of children and adolescents' experiences of pain communication in paediatric rheumatology in the United Kingdom, highlighting the importance and value of these processes to those who experience chronic musculoskeletal pain. Our findings highlight a range of effective and ineffective assessment and communication approaches, which have informed recommendations to improve healthcare professionals' communication about pain in line with children and adolescents expectations and needs.

## AUTHOR CONTRIBUTIONS

All authors were responsible for the conception and study design. JMcD was involved in participant recruitment. RRL performed the data collection, analysis and manuscript writing. DM contributed to the analysis of the data. All authors discussed the results and critically revised the manuscript.

## FUNDING INFORMATION

This work was supported by a Foundation Fellowship award from Versus Arthritis (Grant 22433). Aspects of this work were also supported by funding from the Centre for Epidemiology Versus Arthritis (Grant 20380) and the NIHR Manchester Biomedical Research Centre.

## CONFLICTS OF INTERESTS

All authors declare no conflict of interest.

## Supporting information


Appendix S1

Appendix S2

## References

[ejp2043-bib-0001] Beresford, B. A. , & Sloper, P. (2003). Chronically ill adolescents' experiences of communicating with doctors: A qualitative study. Journal of Adolescent Health, 33, 172–179. 10.1016/s1054-139x(03)00047-8 12944007

[ejp2043-bib-0002] Caplan, R. , & Bursch, B. (2012). How many more questions? Techniques for clinical interviews of young medically ill children. Oxford University Press.

[ejp2043-bib-0003] Chambers, C. T. (2018). From evidence to influence: dissemination and implementation of scientific knowledge for improved pain research and management. Pain, 159, S56–S64. 30113948 10.1097/j.pain.0000000000001327

[ejp2043-bib-0004] Chan, J. Y. , & von Baeyer, C. L. (2016). Cognitive developmental influences on the ability of pre‐school aged children to self‐report their pain intensity. Pain, 157, 997–1001. 26716994 10.1097/j.pain.0000000000000476

[ejp2043-bib-0005] Clinch, J. , & Eccleston, C. (2009). Chronic musculoskeletal pain in children: Assessment and management. Rheumatology, 48, 466–474. 19202161 10.1093/rheumatology/kep001

[ejp2043-bib-0006] Davies, K. , & Copeman, A. (2006). The spectrum of paediatric and adolescent rheumatology. Best Practice & Research Clinical Rheumatology, 20, 179–200. 16546052 10.1016/j.berh.2005.12.002

[ejp2043-bib-0007] Defenderfer, E. K. , Bauer, K. , Igler, E. , Uihlein, J. A. , & Davies, W. H. (2018). The experience of pain dismissal in adolescence. Clinical Journal of Pain, 34, 162–167. 28654555 10.1097/AJP.0000000000000530

[ejp2043-bib-0008] Dell'Api, M. , Rennick, J. E. , & Rosmus, C. (2007). Childhood chornic pain and health care professional interactions: Shaping the chronic pain experiences of children. Journal of Child Health Care, 11, 269–286. 18039730 10.1177/1367493507082756

[ejp2043-bib-0009] Edmond, S. , & Keefe, F. J. (2015). Validating pain communication: Current state of the science. Pain, 156, 215–219. 25599441 10.1097/01.j.pain.0000460301.18207.c2 PMC4477266

[ejp2043-bib-0010] Emerson, N. D. , & Bursch, B. (2020). Communicating with young about pain: Developmental considerttations. Children, 7, 1–12. 10.3390/children7100184 PMC7602494 33076255

[ejp2043-bib-0011] Hadjistavropoulos, T. , Craig, K. D. , Duck, S. , Cano, A. , Goubert, L. , Jackson, P. L. , Mogil, J. S. , Rainville, P. , Sullivan, M. J. L. , Williams, A. C. , Vervoort, T. , & Fitzgerald, T. D. (2011). A biopsychosocial formulation of pain communication. Psychological Bulletin, 137, 910–939. 21639605 10.1037/a0023876

[ejp2043-bib-0012] Harrison, L. E. , Pate, J. W. , Richardson, P. A. , Ickmans, K. , Wicksell, R. K. , & Simons, L. E. (2019). Best‐Evidence for the rehabilitation of chronic pain Part 1: pediatric pain. Journal of Clinical Medicine, 8(9), 1267. 31438483 10.3390/jcm8091267 PMC6780832

[ejp2043-bib-0013] Hirschfeld, G. (2014). Clinically meaningful changes in pain ratings: why we need special cut points in children and adolescents. Pain Management, 4, 81–83. 24641430 10.2217/pmt.14.2

[ejp2043-bib-0014] Huguet, A. , Stinson, J. , & McGrath, P. (2010). Measurement of self‐reported pain intensity in children and adolescents. Journal of Psychosomatic Research, 68, 329–336. 20307699 10.1016/j.jpsychores.2009.06.003

[ejp2043-bib-0015] Igler, E. C. , Defenderfer, E. K. , Lang, A. C. , Bauer, K. , Uihlein, J. , & Davies, W. H. (2017). Gender differences in the experience of pain dismissal in adolescence. Journal of Child Health Care, 21, 381–391. 29110522 10.1177/1367493517727132

[ejp2043-bib-0016] Jordan, A. , Carter, B. , & Vasileiou, K. (2021). "Pain talk". A triadic collaboration in which nurses promote opportunities for engaging children and their parents about managing children's pain. Paediatric and Neonatal Pain, 3, 123–133. 35547948 10.1002/pne2.12061 PMC8975224

[ejp2043-bib-0017] Khanom, S. , McDonagh, J. E. , Briggs, M. , Bakir, E. , & McBeth, J. (2020). Adolescents' experiences of fluctuating pain in musculoskeletal disorders: A qualitative systematic review and thematic synthesis. BMC Musculoskeletal Disorders, 21, 1–16. 10.1186/s12891-020-03627-1 PMC7532580 33008357

[ejp2043-bib-0018] Kim, B. , & White, K. (2018). How can health professionals enhance interpersonal communication with adolescents and young adults to improve health care outcomes?: Systematic literature review. International Journal of Adolescence and Youth, 23(2), 198–218.

[ejp2043-bib-0019] Kimura, Y. , & Walco, G. (2007). Treatment of chronic pain in pediatric rheumatic disease. Nature Clinical Practice Rheumatology, 3, 210–218. 17396106 10.1038/ncprheum0458

[ejp2043-bib-0020] Lang, A. C. , Igler, E. C. , Defenderfer, E. K. , Uihlein, J. , Brimeyer, C. T. , & Davies, W. H. (2018). Evaluating differential effects of specific pain dismissal interactions with physicians. The Clinical Journal of Pain, 34, 664–669. 29298187 10.1097/AJP.0000000000000586

[ejp2043-bib-0021] Lee, R. R. , McDonagh, J. E. , Connelly, M. , Peters, S. , & Cordingley, L. (2021). Identifying the content and context of pain within paediatric rheumatology healthcare professional curricula in the UK: A summative content analysis. Pediatric Rheumatology, 19, 129–140. 34419095 10.1186/s12969-021-00614-1 PMC8379855

[ejp2043-bib-0022] Lee, R. R. , Rashid, A. , Thomson, W. , & Cordingley, L. (2019). “Reluctant to assess pain”: A qualitative study of healthcare professionals' beliefs about the role of pain in Juvenile Idiopathic Arthritis. Arthritis Care & Research, 72(1), 69–77. 10.1002/acr.23827 PMC6973019 30629337

[ejp2043-bib-0023] Lee, R. R. , Rashid, A. , Thomson, W. , & Cordingley, L. (2020). 'Reluctant to assess pain': A qualitative study of healthcare professionals' beliefs about the role of pain in Juvenile Idiopathic Arthritis. Arthritis Care & Research, 72, 69–77. 30629337 10.1002/acr.23827 PMC6973019

[ejp2043-bib-0024] Lefèvre, H. , Loisel, A. , Meunier, B. B. , Deslandre, C. , Lemoine, N. , Moro, M. R. , Quartier, P. , & Lachal, J. (2019). Chronic idiopathic musculoskeletal pain in youth: A qualitative study. Pediatric Rheumatology, 17, 1–9. 31882011 10.1186/s12969-019-0389-3 PMC6935211

[ejp2043-bib-0025] Lincoln, Y. S. , & Guba, E. G. (1985). Naturalistic inquiry. Sage.

[ejp2043-bib-0026] Lundberg, V. , Eriksson, C. , Lind, T. , Coyne, I. , & Fjellman‐Wiklund, A. (2021). How children with juvenile idiopathic arthritis view participation and communication in healthcare encounters: A qualitative study. Pediatric Rheumatology, 19, 1–11. 34727931 10.1186/s12969-021-00642-x PMC8561993

[ejp2043-bib-0027] McGhee, J. L. , Burks, F. N. , Sheckels, J. L. , & Jarvis, J. N. (2002). Identifying children with chronic arthritis based on chief complaints: Absence of predictive value for musculoskeletal pain as an indicator of rheumatic disease in children. Pediatrics, 110, 354–362. 12165590 10.1542/peds.110.2.354

[ejp2043-bib-0028] Rapley, T. , Farre, A. , Parr, J. E. , Wood, V. , Reape, D. , Dovey‐Pearce, G. , McDonagh, J. E. , & The Transition Collaborative Group . (2019). Can we normalise developmentally appropriate health care for young people in UK hospital settings? An ethnographic study. BMJ Open, 9, e029107. 10.1136/bmjopen-2019-029107 PMC6738748 31501109

[ejp2043-bib-0029] Ritchie, J. , & Lewis, J. (2003). Qualitative research practice: A guide for social science students and researchers. Sage.

[ejp2043-bib-0030] Ritchie, J. , & Spencer, L. (1994). Qualitative data analysis for applied policy research. In A. Bryman & R. Burgess (Eds.), Analysing Qualitative Data (pp. 173–194). Routledge.

[ejp2043-bib-0031] Saunders, B. , Sim, J. , Kingstone, T. , Baker, S. , Waterfield, J. , Bartlam, B. , Burroughsm, H. , & Jinks, C. (2018). Saturation in qualitative research: Exploring its conceptualization and operationalization. Quality and Quantity, 52, 1893–1907. 29937585 10.1007/s11135-017-0574-8 PMC5993836

[ejp2043-bib-0032] Schanberg, L. , Anthony, K. , Gil, K. , & Maurin, E. (2003). Daily pain and symptoms in children with polyarticular arthritis. Arthritis and Rheumatism, 48, 1390–1397. 12746912 10.1002/art.10986

[ejp2043-bib-0033] Sørensen, K. , & Christiansen, B. (2017). Adolescents experience of complex persistent pain. Scandinavian Journal of Pain, 15, 106–112. 28850332 10.1016/j.sjpain.2017.02.002

[ejp2043-bib-0034] Sturges, J. E. , & Hanrahan, K. J. (2004). Comparing telephone and face‐to‐face qualitative interviewing: A research note. Qualitative Research, 4(1), 107–118.

[ejp2043-bib-0035] Tong, A. , Sainsbury, P. , & Craig, J. (2007). Consolidated criteria for reporting qualitative research (COREQ): A 32‐item checklist for interviews and focus groups. International Journal for Quality in Health Care, 19, 349–357. 17872937 10.1093/intqhc/mzm042

[ejp2043-bib-0036] Wakefield, E. O. , Zempsky, W. T. , Phul, R. M. , & Litt, M. D. (2018). Conceptualizing pain‐related stigma in adolescent chronic pain: A literature review and preliminary focus group findings. Pain reports, 3(Suppl 1), e679. 30324171 10.1097/PR9.0000000000000679 PMC6172824

