# Supplementary material for: ‘That's what makes me better’: Investigating children and adolescents' experiences of pain communication with healthcare professionals in paediatric rheumatology
Source: Eur J Pain. 2022 Oct 21;27(1):111–28. doi: 10.1002/ejp.2043 (PMC10092465; doi:10.1002/ejp.2043)
Supplement: Supplementary file 1 — Appendix S1 [file EJP-27-111-s001.docx]

**COREQ- (COnsolidated criteria for REporting Qualitative research) checklist (Tong *et al*, 2007)**

| **Topic** | **Item No.** | **Guide questions/Description** | **Reported on Page No.** |
| --- | --- | --- | --- |
| **Domain 1: Research team and reflexivity** | | | |
| ***Personal characteristics*** | | | |
| **Interview/facilitator** | 1 | Which author/s conducted the interview or focus group? | 4/5 |
| **Credentials** | 2 | What were the researcher’s credentials? E.g. PhD, MD | 4/5 |
| **Occupation** | 3 | What was their occupation at the time of the study? | 4/5 |
| **Gender** | 4 | Was the researcher male or female? | 4/5 |
| **Experience and training** | 5 | What experience or training did the researcher have? | 4/5 |
| ***Relationship with participants*** | | | |
| **Relationship established** | 6 | Was a relationship established prior to study commencement? | 4 |
| **Participant knowledge of the interviewer** | 7 | What did the participants know about the researcher? E.g. personal goals, reasons for doing the research | 4 |
| **Interviewer characteristics** | 8 | What characteristics were reported about the interview/facilitator? E.g. bias, assumptions, reasons and interests in the research topic | 4 |
| **Domain 2: Study design** | | | |
| ***Theoretical framework*** | | | |
| **Methodological orientation and theory** | 9 | What methodological orientation was stated to underpin the study? E.g. grounded theory, discourse analysis, ethnography, phenomenology, content analysis | 5 |
| ***Participant selection*** | | | |
| **Sampling** | 10 | How were participants selected? E.g. purposive, convenience, consecutive, snowball | 3/4 |
| **Method of approach** | 11 | How were participants approached? E.g. face-to-face, telephone, mail, email | 3/4 |
| **Sample size** | 12 | How many participants were in the study? | 4/5 |
| **Non-participation** | 13 | How many people refused to participate or dropped out? Reasons? | 4 |
| ***Setting*** | | | |
| **Setting of data collection** | 14 | Where was the data collected? E.g. home, clinic, workplace | 4 |
| **Presence of non-participants** | 15 | Was anyone else present besides the participants and researchers? | 4 |
| **Description of sample** | 16 | What are the important characteristics of the sample? E.g. demographic data, date | 5 |
| ***Data collection*** | | | |
| **Interview guide** | 17 | Were questions, prompts, guides provided by the authors? Was it pilot tested? | 5 |
| **Repeat interviews** | 18 | Were repeat interviews carried out? If yes, how many? | N/A |
| **Audio/visual recording** | 19 | Did the research use audio or visual recording to collect the data? | 5 |
| **Field notes** | 20 | Were field notes made during and/or after the interview or focus group? | N/A |
| **Duration** | 21 | What was the duration of the interviews or focus groups? | 5 |
| **Data saturation** | 22 | Was data saturation discussed? | 5 |
| **Transcripts returned** | 23 | Were transcripts returned to participants for comment and/or correction? | N/A |
| **Domain 3: analysis and findings** | | | |
| ***Data analysis*** | | | |
| **Number of data coders** | 24 | How many data coders coded the data? | 5 |
| **Description of the coding tree** | 25 | Did authors provide description of the coding tree? | N/A |
| **Derivation of themes** | 26 | Were themes identified in advance or derived from the data? | 5 |
| **Software** | 27 | What software, if applicable, was used to manage the data? | 5 |
| **Participant checking** | 28 | Did participants provide feedback on the findings? | N/A |
| ***Reporting*** | | | |
| **Quotations presented** | 29 | Were participant quotations presented to illustrate the themes/findings? Was each quotation identified? E.g. participant number | 6/7/8/9/10/11/12/13/14/15 |
| **Data and findings consistent** | 30 | Was there consistency between the data presented and the findings? | 15 |
| **Clarity of major themes** | 31 | Were major themes clearly presented in the findings? | 6 |
| **Clarity of minor themes** | 32 | Is there a description of diverse cases or discussion of minor themes? | 6/7/8/9/10/11/12/13/14/15 |
